# Combining Machine Learning and Backgrounded Membrane Imaging: A case Study in Comparing and Classifying Different types of Biopharmaceutically Relevant Particles

**DOI:** 10.1016/j.xphs.2022.05.022

**Published:** 2022-06-01

**Authors:** Christopher P. Calderon, Ana Krhač Levačić, Constanze Helbig, Klaus Wuchner, Tim Menzen

**Affiliations:** a Ursa Analytics, Inc., Denver, CO 80212; b Department of Chemical and Biological Engineering, University of Colorado Boulder, Boulder, Colorado 80303, United States; c Coriolis Pharma Research GmbH, Fraunhoferstr. 18 b, 82152 Martinsried, Germany; d Janssen Research and Development, DPDS BTDS Analytical Development, Hochstr. 201, 8200 Schaffhausen, Switzerland

**Keywords:** Biopharmaceutical characterization, Image analysis, Imaging methods, Morphology, Protein aggregation, Monoclonal antibody(s), Surfactant(s), Protein formulation, Microparticle(s)

## Abstract

This study investigates how backgrounded membrane imaging (BMI) can be used in combination with convolutional neural networks (CNNs) in order to quantitatively and qualitatively study subvisible particles in both protein biopharmaceuticals and samples containing synthetic model particles. BMI requires low sample volumes and avoids many technical complications associated with imaging particles in solution, e.g., air bubble interference, low refractive index contrast between solution and particles of interest, etc. Hence, BMI is an attractive technique for characterizing particles at various stages of drug product development. However, to date, the morphological information encoded in brightfield BMI images has scarcely been utilized. Here we show that CNN based methods can be useful in extracting morphological information from (label-free) brightfield BMI particle images. Images of particles from biopharmaceutical products and from laboratory prepared samples were analyzed with two types of CNN based approaches: traditional supervised classifiers and a recently proposed fingerprinting analysis method. We demonstrate that the CNN based methods are able to efficiently leverage BMI data to distinguish between particles comprised of different proteins, various fatty acids (representing polysorbate degradation related particles), and protein surrogates (NIST ETFE reference material) only based on BMI images. The utility of using the fingerprinting method for comparing morphological differences and similarities of particles formed in distinct drug products and/or laboratory prepared samples is further demonstrated and discussed through three case studies.

## Introduction

Particulate matter is considered a critical quality attribute in the development and manufacturing of biopharmaceutical drug products. Particles found in drug products can originate from environmental contamination (unexpected foreign particles), manufacturing process (e.g., filter material, stainless steel), excipients (e.g., free fatty acid particles), active pharmaceutical ingredients (API) like therapeutic proteins (e.g., protein particles), or can be container related (e.g., silicone oil).^1^

The occurrence of proteinaceous particles and aggregates in biopharmaceuticals may result in unintended immunological effects,^1,2^ and thus may pose a safety concern.^3–5^ Protein particle formation can be induced by different chemical^6^ or physical degradation^7^ mechanisms induced by numerous stress factors (e.g., temperature excursions, interfacial or mechanical stress).^8–10^

The non-ionic surfactants polysorbates 20 and 80 (PS20, PS80) are the most commonly used stabilizers in biopharmaceuticals and are contained in >70% of marketed parenteral biological drug products.^11^ Polysorbates improve product shelf life by their ability to protect protein against interfacial stress, to prevent protein adsorption to container surfaces, and to stabilize protein against stresses during common manufacturing processes (e.g., fill/finish, lyophilization).^2,12–14^ Polysorbates exhibit great structural heterogeneity, which is caused by the diversity in fatty acid ester distribution, polyoxyethylene chain length, and core sugar structure.^13,15^ During storage of liquid drug product, enzymatic hydrolysis or oxidation of polysorbate can occur, which can result in the release of degradation products such as free fatty acids (FAs). FAs are poorly soluble in aqueous solutions and can form visible and/or subvisible particles.^2,4,5,13,14,16–18^

Both proteinaceous and non-proteinaceous particles in the visible and subvisible size range need to be analyzed to monitor the stability of therapeutic protein formulations. One potential particle characterization technique is backgrounded membrane imaging (BMI), an automated, 96-well plate based approach for the quantification and characterization of subvisible particles >2 μm. BMI offers high throughput analysis as well as low sample consumption.^19^ Since analysis of isolated particles on the membrane surface occurs in air, BMI does not suffer from interferences by air bubbles and is insensitive to the formulation’s refractive index.

Discrimination and classification of particles in a protein drug product or distinguishing particles from various distinct formulations are demanding because particles exhibit a high degree of heterogeneity in origin, size and morphology.^1^ For example, characterization and quantification of FA particles resulting from degradation of polysorbate are challenging due to the fact that, by human eye, FA particles often exhibit similar morphology as other particle types (e.g., protein aggregates). Unfortunately, most of the morphological information contained in particle images is usually not exploited during data analysis, e.g., only a small number of simple morphological features related to size and shape is utilized in standard BMI analysis. A deeper qualitative insight into collected (BMI) particle images should be possible when data analysis is complemented by artificial intelligence (AI) methods.

In recent years, AI has demonstrated substantial progress in the analysis of particle images. Through its ability to extract data features from imaged particles, AI can leverage subtle morphology differences to discriminate particle populations and enables new particle classification and characterization approaches. Such classification methods have already been demonstrated on images from flow imaging microscopy.^20–22^ In particular, it has already been observed, by convolutional neural network (CNN) based methods, that signatures of various stresses (e.g., mechanical agitation or temperature shocks) can manifest as distinct morphologies in protein aggregates imaged by flow imaging microscopy.^20,21,23–25^ Accordingly, AI based approaches such as CNNs have great potential to support and supplement the analysis of collected (BMI) particle images. However, it was unknown to the authors prior to this study, if the isolation of particles on a membrane surface including vacuum application necessary in BMI would compromise the morphological information of various particle types encoded in digital images that has been successfully used in previous flow imaging based approaches.

Although CNN based classification can be extremely accurate in identifying particles, these classifiers exhibit a major inherent limitation in their inability to identify particles formed under stress conditions not covered by the underlying CNN training data (the potential aggregation-inducing stresses must be known *a priori*). In contrast to traditional supervised CNN based classifiers, the fingerprinting approach can identify when new “classes” are encountered (i.e., the method can detect novel particle populations not contained in the training data). With the fingerprinting approach, a high dimensional image is compressed to a two-dimensional (2D) image representation we refer to as an “embedding”.^26,27^ The fingerprinting approach was originally motivated as a dimension reduction technique aimed at quality control applications (where extreme dimension reduction is necessary for nonparametric density estimation and formal goodness-of-fit testing).^20^ In our present study, we focus on showing how the fingerprinting method can be used to compare heterogeneous particle populations.

Specifically, in this study, we used BMI, to generate images of subvisible particles from three different groups of samples containing a variety of particles including: protein based drug products (DP A, DP B, and DP C), synthetic model FA particles (from a single FA or from a mixture of FAs) and ethylene tetraflouroethylene (ETFE) particles, a NIST protein particle surrogate. Our main purpose was to develop and evaluate automated image analysis methods based on CNNs for the comparison and classification of images from different types of pharmaceutically relevant particles. In addition, the potential impact of BMI-specific sample preparation (vacuum application, drying on a membrane surface) on the particle morphology information and consequently on the possibility of distinguishing different types of particles was evaluated. Going beyond typical CNN based classification, the recent fingerprinting approach^20^ was evaluated to not only compare particle classes/types but also improve studies on subvisible particle formation mechanisms in drug products. We also explore how the fingerprinting approach can allow for a more quantitative comparison of similarities and differences between different particle types.

## Materials and Methods

### Materials

Drug products (DPs) contained 20 to >100 mg/mL IgG1-type monoclonal antibodies (mAbs) formulated at a pH between 5-6 in different buffers (Table 1). The DPs were selected due to the presence of well characterized product related particles. DP A was stored at −80°C, DP B and DP C were stored at 2-8°C until the day of BMI analysis.

FAs used to prepare synthetic model FA particles (Table 1) were of analytical standard grade and obtained from Merck (Darmstadt, Germany). Excipients used for the preparation of formulation buffers were of “Ph. Eur.” or better grade. Highly purified water was used throughout the study (Milli-Q® IQ 7000 purification system; Merck).

ETFE particles (Reference Material 8634) were obtained from the National Institute of Standards and Technology (Gaithersburg, MD, United States).

### Generation of FA Particles as Model for Particles Originating from Polysorbate Degradation

Details on FA sample composition are summarized in Table 1. For particle samples containing a single FA species, the respective FA was dissolved at a 1,000-fold of the target concentration in 2-propanol. 2-Propanol was filtered with a 0.22-*μ*m hydrophilic polyvinylidene fluoride (PVDF) syringe filter (Merck) prior to sample preparation. Dissolved FA was then spiked into a formulation buffer of same composition as for DP C in a 6R glass vial. Particle samples were homogenized by inversion and incubated for 6 to 7 days at 2-8°C. For particle samples containing multiple FA species, stock solutions each containing a single FA were prepared in filtered 2-propanol at a 7,000-fold of the target concentration of the respective FA. A stock of the FA mixture in 2-propanol was then obtained by mixing the single FA stocks at equal volumes. The mixed FA stock was then spiked into formulation buffer as described above. Samples Mix I and Mix II were incubated at 2-8°C up to 5.5 months. Sample Mix III, containing additional salts, was incubated at 2-8°C and after incubation times of 36 hours and 1, 4, and 5.5 months sample volumes for BMI analysis were withdrawn from the same vial after homogenization.

### Particle Analysis by Backgrounded Membrane imaging (BMI)

BMI analysis was performed with a HORIZON instrument (Halo Labs, Philadelphia, PA, USA). Application of samples to 96-well membrane plates (Halo Labs) was carried out in a laminar air flow cabinet. A volume of 30 - 420 *μ*L of sample was pipetted per well and vacuum was applied at 350 mbar below ambient pressure to remove liquid from the membrane plate. In the case of samples where more than 60 *μ*L of sample per well was required, the sample was pipetted in successive steps of 60 *μ*L with vacuum suction after each step. After sample application and vacuum suction, each well was washed with at least the 1.5-fold volume of water.

### Characterization of Nature of Particles in Protein Based Drug Products

Particles present in DP A to C were characterized after isolation on gold-coated membrane filters by micro-spectroscopic techniques. Scanning electron microscopy coupled to an energy dispersive X-ray detector (SEM-EDX) allowed a semi-quantitative analysis of the elemental composition of particles. FTIR measurements in attenuated total reflection mode (ATR-FTIR) in a spectral range of 4000-600 cm^−1^ were performed on isolated particles to determine their organic nature. In addition, isolated DP C particles were further characterized by liquid chromatography mass spectrometry (LC-MS) to determine in greater depth their organic nature qualitatively and quantitatively.

### Computational Details for Analyzing BMI images via CNNs

The classification and embedding analyses were performed on BMI particle image sets without any detailed information on the type or composition of particles, or on applied experimental protocols (the samples were blinded and denominated as sequentially numbered particle images).

The approach outlined in Daniels et al. (2020)^25^ was followed for CNN model construction. The specific CNN network architecture used for training the classification algorithm is provided in Fig. S1; a minor variant of this network architecture was used for the CNN based embedding used in fingerprinting (see Fig. S1 caption for details). Below, we outline the main steps of the CNN-based analysis.

### BMI image Pre-Processing

Images of individual particles, denoted as collected BMI particle images, were extracted from the HORIZON instrument’s background-corrected well-images using proprietary software. The neural network was configured to process grayscale images that were 32 × 32 pixels; the results in this work were found to be insensitive to the precise pixel size (a comparison of output obtained using 24 × 24 images is reported in the supporting information). In order to achieve this size and keep the spatial image resolution fixed throughout (i.e., no image rescaling applied), the following pre-processing steps were implemented before inputting the images to the neural network: for individual particle images smaller than 32 pixels in either dimension, image borders were extended with a constant intensity (the extended pixels took on the average pixel intensity of the training set) with the original image centered within this padded border to achieve the target image size (black borders and other image padding strategies were also tested and generated nearly identical results); for individual particle images larger than 32 pixels in either dimension, the particle images were centered and cropped to the target size used in the CNN network. A total of N ≈ 140,000 collected (BMI) particle images was available to be analyzed by two primary types of CNN models considered in this work: CNN classification networks and closely related CNN embedding networks. The various CNNs used in different sections differ primarily in the training data used to calibrate the models and/or the loss objective function used to estimate CNN models; all images were subsequently normalized by subtracting the empirical training set pixel intensity mean and subsequently dividing by the training set pixel intensity standard deviation.

### Classification Networks

For CNN based classification models, the extracted individual images with a label, i.e., particle type information, used explicitly in the model were shuffled and randomly assigned to the train or test set (80/20% train/test split, where 80% were used for training and 20% were used as test data). Test data were not used in training the CNN model. Image sample sizes used for each CNN are reported in the Results and Discussion. The classification network (see Fig. S1 for details) was trained for 15 epochs (an epoch is one complete pass through the training data) using an Adam optimizer with the AMSgrad option set to true with a minibatch size of 256 (all other parameters were default) optimizing a weighted cross entropy loss objective function. In all classification models studies, weights were selected to be inversely proportional to the number of samples of each category.

### Embedding Networks

The embedding network architecture differs from the classification network only in the last layer (see Fig. S1). However, a different objective loss function is used to estimate the CNN parameters from the training data. The “Batch All non-zero” triplet loss objective function^26^ (using 32 images of each class to construct triplet minibatches) with squared Euclidean distances (and margin hyperparameter = 1.0) was used as the loss function for obtaining the CNN parameters of the embedding network. The CNN embedding network was trained with the same optimizer using an 80/20% train/validation set split. The validation set was used to monitor the loss function with a “patience” of 5 (i.e., the parameter optimization was ceased if the validation loss function did not decrease beyond the empirically observed minimum loss function value for 5 epochs). The CNN parameters yielding the minimum triplet loss on the validation set were selected in an effort to avoid over-fitting.

### Probability Density Function (pdf) or Fingerprint Estimation and Goodness-of-fit Statistic Computation

The scatterplot of CNN embeddings for a given class of interest was used to derive the corresponding probability density function (pdf); this is what we refer to as a fingerprint. To compute the pdf or fingerprint, the CRAN package ks^28^ was leveraged to obtain the optimal general bandwidth matrix (with off-diagonal terms) using the plug-in matrix bandwidth selector associated with a Gaussian kernel density estimate (the data and optimal bandwidth then permit evaluation of the pdf at any point of interest). Due to (relatively) low particle numbers in the studied cases, the entire dataset was used to construct the fingerprints (in contrast to the approach taken in Calderon et al.^25^).

For goodness-of-fit hypothesis testing, a reference fingerprint of interest was selected as the null pdf; the null hypothesis is that the observed data represents random samples drawn from the null pdf and the alternative hypothesis is that the observed data are random samples drawn from any other pdf. Since real world FA particles due to polysorbate degradation were one of our primary interests, in this work, we used collected (BMI) particle images of DP C as the null reference pdf and tested images from all other samples (e.g., synthetic model FA particles). Using the null pdf and scatterplot data from a class of interest, the Rosenblatt transform^29^ was computed using custom Python functions. The Kolmogorov-Smirnov goodness-of-fit test statistic^30^ was then computed using the function kstest in the stat package of scipy (version 1.4.1). To address over-rejection (i.e., the practical vs. statistical significance problem^31^), we divided each test scatterplot data set into *N_test_* = 100 subsets and applied the goodness-of-fit test on samples of size *N_test_* (the net data was randomly shuffled without replacement and grouped into subsets of size *N_test_*) and the percent of subsets rejected was recorded. For example, if a given class had a total of 30,000 images, 300 = 30,000/*N_test_* subsets would be formed and the fraction of rejected goodness-of-fit tests in that collection of tests is used to quantify the proximity of the consistency/inconsistency of the embedding scatterplot data with the selected null pdf. A low fraction rejection indicates statistical consistency and a high fraction rejection indicates an inconsistency with the null pdf.

### Computational Environment Details

All computations (CNN and goodness-of-fit analyses) were carried out in Python 3.7.1 using Pytorch 1.7.0 in a Docker container running Ubuntu 18.04.5 LTS connected to two Nvidia GeForce 1080s.

## Results and Discussion

### Classification of Collected BMI Particle Images into Different Classes (FA, Protein, or ETFE Particles)

For the proof-of-concept study, we analyzed particle images from (i) the palmitic acid (PalA) particle sample, (ii) two drug product samples (DP A, DP B) containing protein particles, and (iii) ETFE particles in order to create four classes of particles from fundamentally different sources (Table 1). Particles in DP A and DP B were identified as proteinaceous particles with no meaningful levels of other constituents via ATR-FTIR and SEM-EDX (using an experimental approach similar to Cao et al. (2015)^4^). Besides being related to different proteins and protein concentrations, formation mechanism and age of particles in DP A and B are also different: protein particles in DP A were generated on a short time scale due to fill-finish stress, whereas DP B protein particles were slowly formed over years (>5 years) during long-term storage at 2-8°C. Hence, we aimed to see if BMI images of proteinaceous particles in DP A and DP B could be distinguished in the first classifier considered. ETFE particles are abraded polymer particles which are time-stable and have similar optical properties relative to protein aggregates in solution. The abraded ETFE studied in this work was developed by NIST as a protein surrogate originally for size and count applications in flow imaging microscopy.^32^ We wanted to study if the morphology of this surrogate protein-particle standard exhibits morphologies of protein particles when imaged by BMI.

**Figure 1.**
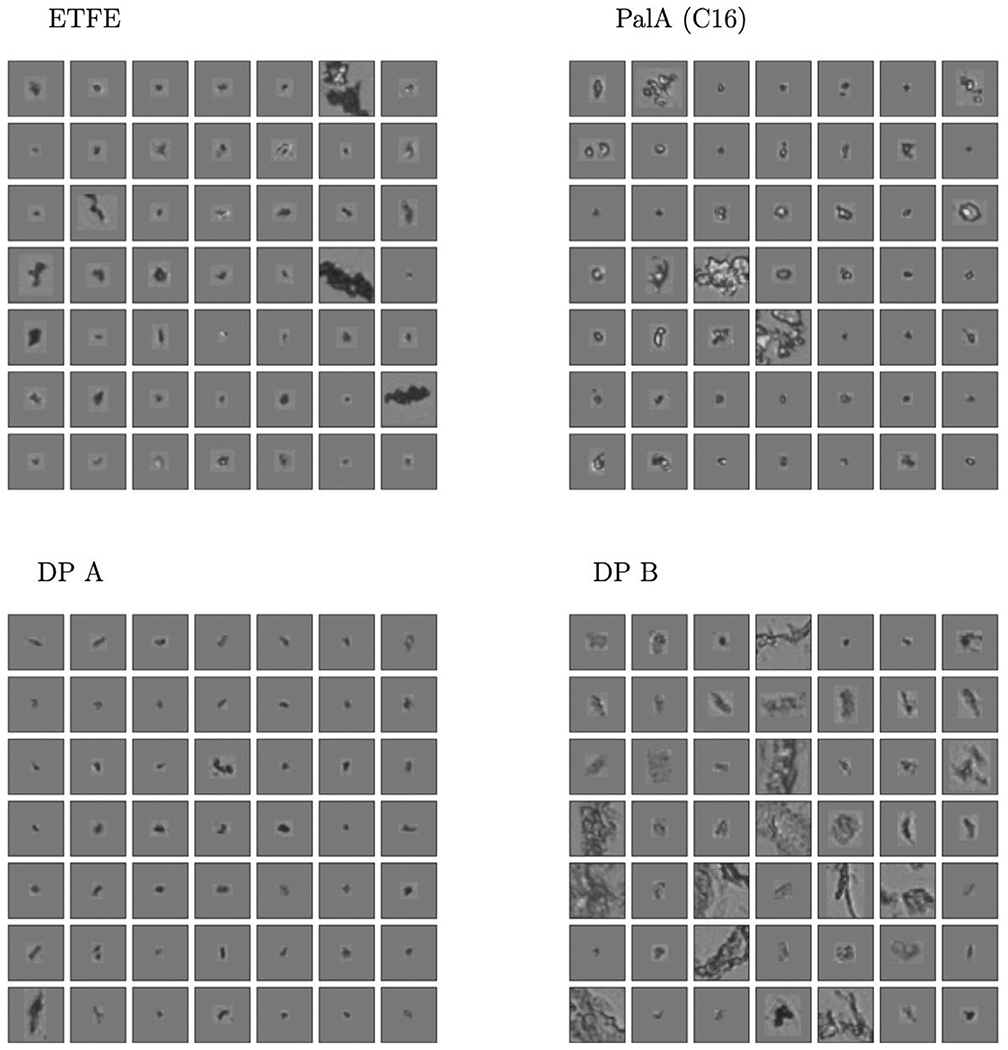
Randomly selected particle BMI images of ETFE, PalA (C16), and DP A and DP B.

Figure 1 shows randomly selected particle images from the four classes in the first CNN based classification model considered; images of PalA particles, protein particles in DP A or DP B, or ETFE particles are displayed in each panel. In addition, Table 2 displays the test classification accuracy achieved by this traditional supervised CNN model (see Fig. S1 for neural network details) for the four classes considered explicitly in training the CNN model of this section. The numbers reported in the table correspond to the fraction of correctly labeled images for the test data. The neural network was trained with a total of ≈ 75,000 images of the four classes split into an 80/20% train/test set. Note that the sum of the rows always equals one in the classifiers studied.

Table 3 focuses on test results obtained via a different CNN model; this neural network was trained only with BMI images of different FA particle samples (trained with ≈75,000 images split evenly into an 80/20% train/test set). The confusion matrix summarizes the classification results obtained with the 20% test data comprising of particles generated with single FAs (C12 to C18) or with a mixture of FAs at two different concentrations (see Table 1).

Finally, in Table 4 we show classification results for BMI particle images from DP C with the neural network applied in Table 3. Roughly 20,000 BMI images of this out-of-sample particle type (i.e., no images of the DP C class were used in training or testing) were classified into the predefined FA particle classes of the CNN. The particles in DP C were characterized by LC-MS as FAs or salts of FAs with no significant amount of protein (see section 2.4) and originated from enzymatic degradation of polysorbate.

### Discussion

The randomly selected particle images in Fig. 1 display that both dramatic and subtle morphological differences exist in BMI particle images sampled from the four classes summarized in Table 2. Table 2 demonstrates that BMI images contain enough morphological information about the underlying particles to accurately distinguish between particles from the FAs, two proteins, and protein surrogates. BMI particle images originating from a given sample were classified as distinct population and being different to particles from other samples/types. ETFE particle images were different from protein particle images in two included DPs (differing in protein, protein concentration, formulation, particle formation mechanism, particle age), and particle images of synthetic model FA particles generated by spiking palmitic acid into a placebo formulation. The accuracy achievable by a standard CNN classifier using BMI data is encouraging. For example, 77% of the PalA particle images and 81% of the proteinaceous particle images in DP A as well as 82% of the proteinaceous particle images from DP B were correctly classified. Similarly, ETFE could be differentiated in 95% of the test images.

The classification accuracy achievable is notable since the imaged particles are not in their native solution environment when analyzed by BMI. Prior to this study, it was unknown to the authors if the isolation of particles on a membrane surface with applying vacuum and drying steps inherent in the BMI method would sufficiently preserve the morphology of different particle types or origins.

The previous result encouraged us to evaluate if BMI images of particles of various FAs (placebo spiked with FA of a chain length from C12 to C18) and FA mixtures at two different concentrations were similar or resulted in distinct morphologies. As can be observed in Table 3, the BMI FA particle images were distinguished by the traditional classification network. Note that in this case there are six classes in the CNN model, so a random classifier would predict the correct answer 1/6 of the time. The diagonal entries are the largest in each test case and greatly exceed the random guess fraction (i.e., 1/6 = 0.17), showing that even BMI particle images from a large collection of FAs exhibit distinct morphology detectable by a CNN. These results indicate that FAs of variable carbon chain lengths form particles with distinct image signatures detectable by CNNs under our experimental conditions. When generated from a mixture of FAs, subtle systematic differences in particle morphology (that can be seen by careful eye inspection) are detected for samples Mix I and Mix II by CNNs, despite the two mixtures differing only in FA concentration. This could be partly explained by solubility limits of the applied FA species in aqueous solutions. The solubility limits of the three major FAs (out of the seven FAs contained in Mix I and Mix II, see Table 1) at 2-8°C were determined by Doshi et al. (2015)^2^ in a formulation containing 0.04% (w/v) PS20 at pH 5.7. The solubility limits of lauric, myristic, and palmitic acid are 19 ± 1 *μ*g/mL, 3 ± 1 *μ*g/mL, and 1.5 ± 0.5 *μ*g/mL, respectively. In FA Mix II (11 *μ*g/mL) the concentrations of palmitic (1.3*μ*g/mL) and myristic acid (2.0 *μ*g/mL) were below their solubility limits and the particles appeared slightly brighter and less dense compared to FA Mix I (22 *μ*g/mL) (see Fig. S3). In FA Mix I with high FA concentration, the concentrations of palmitic (2.5 *μ*g/mL) and myristic acid (4.0 *μ*g/mL) exceeded the solubility limits and the particles appeared slightly darker. Accordingly, understanding the solubility limits of FAs in pharmaceutically relevant solutions is crucial not only for understanding the reasons for nucleation and precipitation resulting in particle formation (see Section 3.2.2 for additional discussion), but also for understanding the morphological differences of the formed particles. It would certainly be useful to know the solubility limits of all seven FAs contained in the mixed FA samples to further understand the results obtained. Nevertheless, these results suggest that the CNN was able to detect subtle differences in morphology of FA particles originating from changes only in the total concentration of FAs.

Table 4 shows that PalA (C16) particles are the closest match when the CNN model, trained on the synthetic model FA particles shown in Table 3, is forced to classify DP C particles into one of the six classes used in CNN training. In particular, for the FA related particles in DP C, which formed due to enzymatic degradation of polysorbate, more than half of the images were classified by the neural network as being most similar to PalA followed by SteA particles and particles in FA Mix I (see Table 4). Interestingly, LC-MS analysis indicates that particles in DP C contain 10% stearic acid, 33% palmitic acid, and 45% myristic acid (see Section 3.2.2 for further discussion and analysis). However, a classifier match on an out-of-sample test case (recall the CNN classifier was only trained on synthetic model FA particle classes and not DP C) does not indicate identical particle types. In the next section, we explore how the fingerprinting method can be used to more quantitatively explore the similarities and differences of various particle types with particles of DP C.

### Qualitative and Quantitative Comparison of BMI Particle Images by Fingerprint Analysis

In this subsection, we evaluate the fingerprinting approach as an alternative to supervised CNN classification for the qualitative and quantitative comparison of particle types coming from a variety of conditions in three exemplary case studies. The case studies aim at gaining insights about protein particles formed by different mechanisms in different DPs (case study 1); studying particles from different FAs in laboratory samples and formed in DP due to PS20 degradation (case study 2); characterizing morphological changes of synthetic model FA particles over time (case study 3). All samples studied throughout are presented in Table 1.

**Figure 2.**
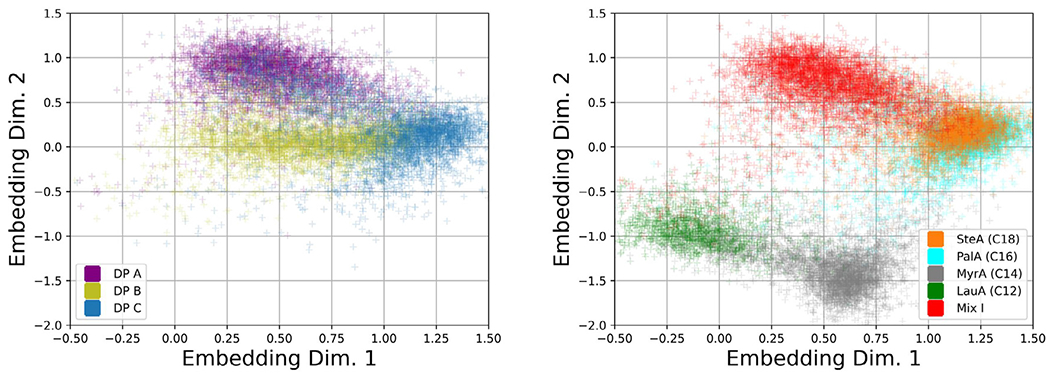
Embeddings of DPs and FAs. A neural network was trained to create an embedding which maps the grayscale pixel intensities from a labeled BMI image collection into a 2D scatterplot representation (i.e., we perform dimension reduction). Shown are the embedding data used to create probability density function (pdfs), referred to as fingerprints. Embeddings of some samples overlap substantially in embedding space due to similar particle morphologies, hence we have separated the embeddings into two groups of plots. In addition, fingerprint contour plots of selected samples shown in Fig. 3 further aid in illustrating the similarities and differences in overlapping fingerprints.

The embeddings, i.e., the scatterplot of dimension reduced representations of BMI particle images, used in the fingerprint method based on six samples containing FA particles (including DP C) and two protein particle samples (DP A and DP B) are shown in Fig. 2. Since many of the embeddings overlap, the embeddings are split into two separate plots in Fig. 2 to improve clarity (however all 8 classes were simultaneously used in training the CNN embedding network). Despite the substantial dimensional reduction of the BMI particle images into a 2D embedding space, the fingerprinting approach still reveals differences in particle morphologies.

Recall that the CNN embeddings used in our fingerprint analysis utilize a nonlinear CNN whose parameters were obtained by maximizing an objective function that explicitly leverages user-supplied training image label information (here “labels” correspond to the Sample Designations shown in Table 1) in order to create a lowdimensional embedding with nonlinear function approximation. Principal components analysis (PCA) is a linear unsupervised dimension reduction technique; traditional PCA aims at using unlabeled data in order to construct an orthonormal basis capable of approximately reconstructing the entire image with a subset of the obtained basis vectors. In Fig. S2, we show PCA embeddings obtained by analyzing the same data shown in Fig. 2a. In contrast to the CNN embeddings, the embeddings of DP A to C obtained using PCA overlap heavily for a majority of the drug product samples (although each PCA embedding point cloud exhibits a slightly different shape). Comparing Fig. 2a and Fig. S2, one can observe a benefit of a CNN dimension reduction technique utilizing labels.

### Case Study 1: Comparison of BMI Particle Images for Protein Particles Formed by Different Mechanisms in Different DPs

**Figure 3.**
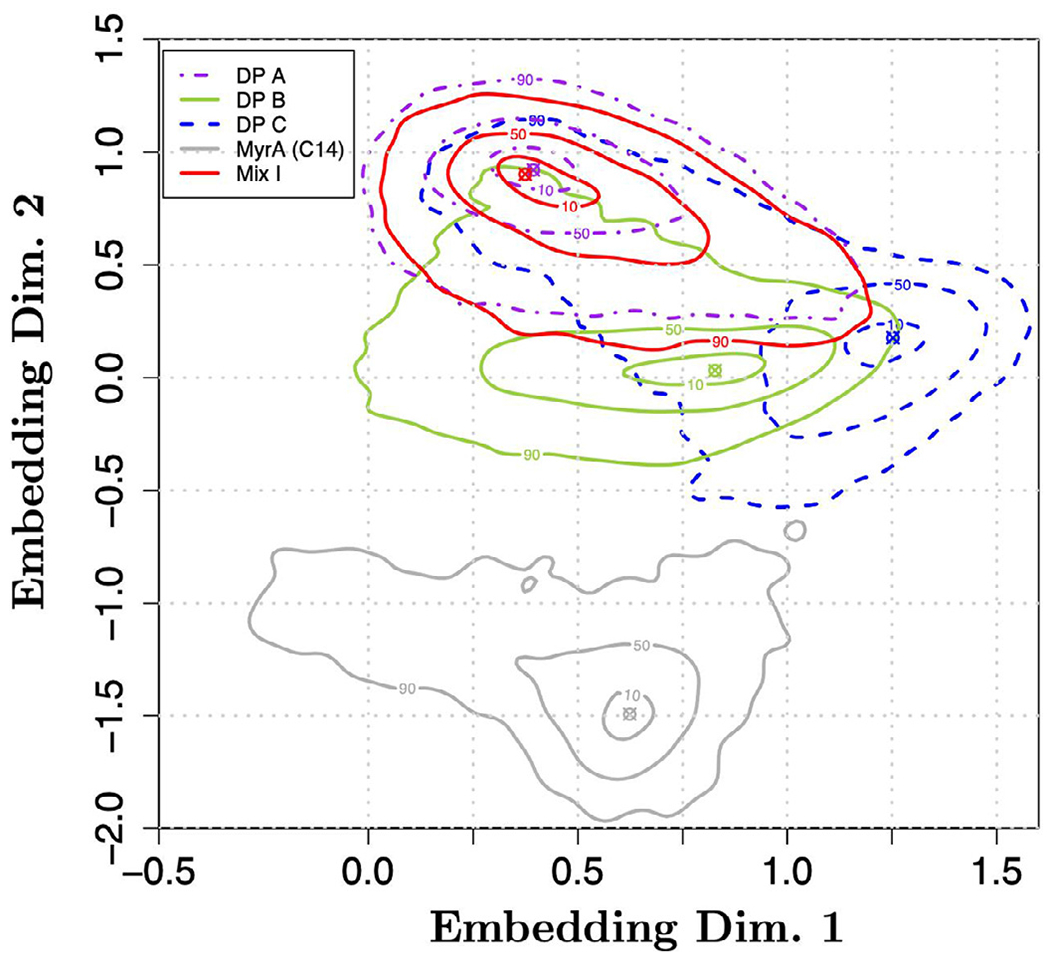
Probability density functions (pdfs) or fingerprints of embeddings coming from five selected particle types. The fingerprints were estimated from the embeddings shown in Fig. 2. The contour levels displayed correspond to the probability mass covered by the enclosed area; the mode of the distribution (indicated by circles with x’s for each case) is in the middle of the lowest level set shown and each of the other levels displayed is the area associated with the %-value of the probability mass (e.g., 80% indicates the region containing 80% of the probability mass centered at the mode).

In Fig. 3, fingerprints, i.e., estimated nonparametric pdfs, approximating the distribution of embeddings of selected representative cases from Fig. 2 are shown. In particular, Fig. 3 compares the fingerprints for DP A and DP B (the two drug products with proteinaceous particles) against DP C, MyrA and FA Mix I in order to illustrate two items: (i) embedding points corresponding to distant regions exhibit distinct morphologies as judged by a human observer; and (ii) images belonging to different classes and exhibiting similar fingerprints (e.g., FA Mix I and DP A) also tend to have very similar particle morphologies.

**Figure 4.**
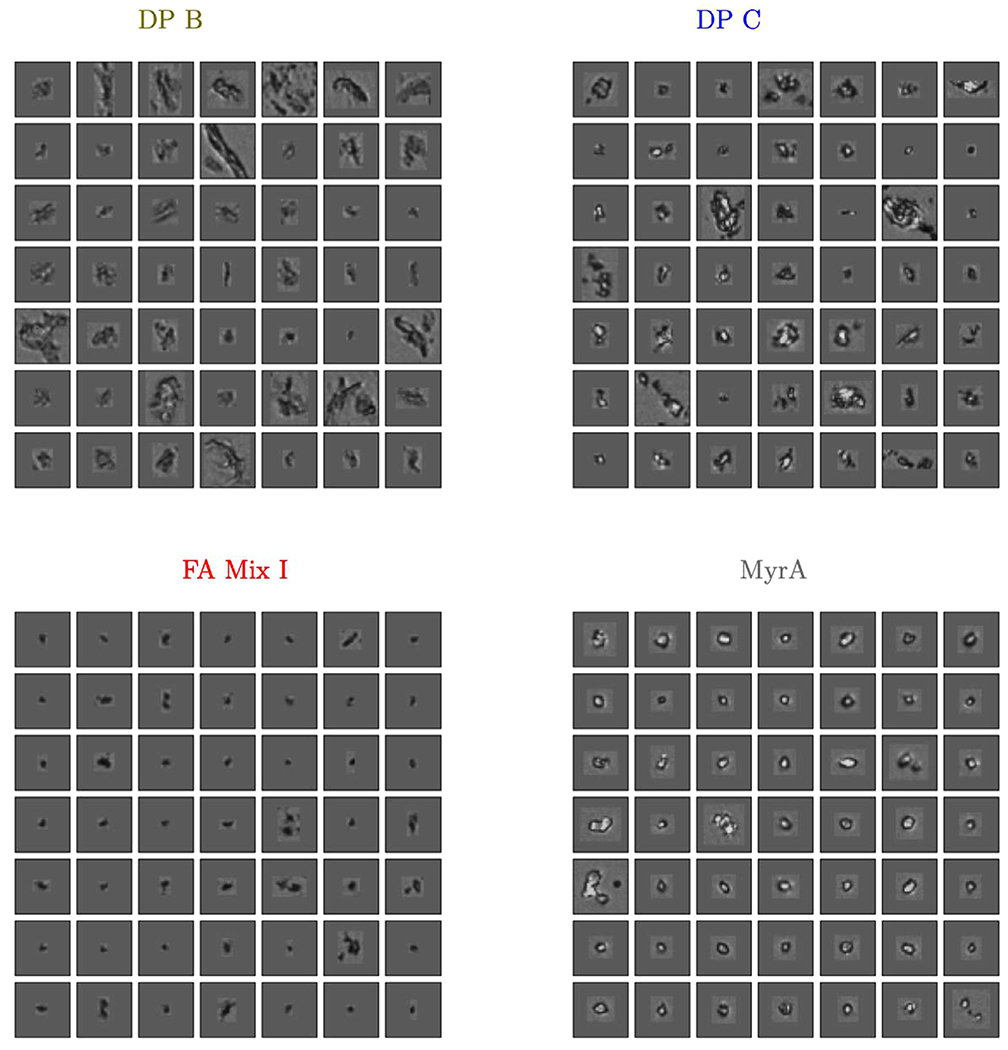
Source BMI images nearest four of the modes shown in Fig. 3. Note how the spatially distant regions of Fig. 3 correspond to distinct particle morphologies. Fingerprints of FA Mix I and DP A overlap globally to a high-degree and particle images from FA Mix I and DP A appear to show similar particle morphologies (randomly sampled DP A particles are shown in Fig. 1 and exhibit similarity to typical FA Mix I particles shown here).

To illustrate both of these points, the ≈ 50 closest (in terms of Euclidean distance) embedding points of DP B, DP C, MyrA and FA Mix I to their corresponding global pdf modes (i.e., the global maximum of the pdf denoted by circled x’s in Fig. 3) were computed and corresponding images are presented in Fig. 4.

### Discussion.

Four cases with distinct pdf modes exhibit distinct morphologies as can be observed by inspecting Fig. 4. It is worth explicitly noting that the particle populations in the two different DPs with proteinaceous particles (DP A, DP B) exhibited readily distinguishable fingerprints with well separated modes (Fig. 3) indicating that the BMI images of the protein particles generated by different mechanisms can be separated using the fingerprinting approach.

Whereas the CNN classifier in the previous section was comprised of 32 dimensions, the features from the BMI images are compressed into a 2D embedding in the fingerprint. This strong dimension reduction might reduce separability compared to a CNN classifier, but the embedding representation often appears to “curate” a highly heterogeneous particle collection into representative particle images. Consequently, the curation enables the observation of common texture and structure compared to randomly selected images. For instance, particles in images in the pdf mode of DP B, DP C, and MyrA look differently in terms of shape and brightness (Fig. 4) and the modes are also well separated in the embedding space (Fig. 3). Nevertheless, the fingerprinting approach was unable to create a 2D representation that could separate DP A particle images from FA Mix I images (while simultaneously encoding information from the six other particle types shown in Fig. 2). This is likely due to the high degree of similarity of FA Mix I (see Fig. 4) and DP A particle images (see Fig. 1) which indicates that different particle types might not always exhibit sufficiently different morphologies for discrimination by the fingerprinting approach. As mentioned, the algorithm is aiming to compress the BMI particle image data down to just two numbers, and if fingerprints are close in shape, the underlying particle morphologies are likely similar. Furthermore, when comparing Fig. 3 and 4, the results of this case study illustrate that embeddings derived from BMI images (which avoid solution based refractive index contrast issues) can also correlate with human interpretable morphologies. However, there is no guarantee that embeddings computed by CNN loss functions correlate with human interpretable features or “morphologies” such as shape and brightness. In contrast, CNN computed features can encode information at diverse length scales,^33^ which may not be obvious to human interpretation. In line with our present findings made for BMI images, we have empirically observed embeddings from CNN and human interpretable features correlate in flow imaging microscopy in previous studies.^20,24,25^ That is, the CNN based embedding scheme is not likely leveraging subtle run specific image differences induced by, e.g., variations in illumination or drifting optics. However, we would like to explicitly note that the fingerprinting algorithm can be used to embed samples not considered in the training scheme. If the resulting fingerprints are similar (or different) one can have confidence that the underlying particle morphologies are similar (or different) in the corresponding BMI images. In previous work^25^, we demonstrated how the fingerprinting approach can detect novel particle populations. In this work, we focus on using the fingerprinting approach to compare particle morphologies from different drug products and synthetic model FA particle samples.

### Case Study 2: Comparison of BMI Particle Images of FA Particles in a DP with Laboratory Prepared FA Model Particle Samples

In this case study, the same CNN embedding (fingerprinting) network shown in the previous section is utilized to qualitatively and quantitatively compare similarity of embeddings corresponding to BMI images of FA particles originating from polysorbate degradation in DP C and of synthetic model FA particles (see Table 1). The comparison aims to determine the suitability of using different synthetic model FA particles, which can be generated in the time scale of hours to days, to mimic FA particles formed as a consequence of polysorbate degradation within a time frame of several months.

Similarity of BMI images of the synthetic model FA particles to the BMI particle images from DP C was quantitatively determined by the goodness-of-fit hypothesis testing described in the Methods. For this purpose, the DP C pdf was used as the null density and the various laboratory prepared FA particle samples as well as DP A and DP B (both representative for pharmaceutically relevant protein particles) were tested to see how similar these cases are to DP C. The class with the lowest rejection rate was deemed the closest match to the FA particles in DP C. When subjected to the stringent goodness-of-fit test obtained using DP C as the null pdf, with N=100 particle image subsets, DP A, DP B and all FA samples were rejected at 100% except PalA and SteA. SteA particle images exhibited the second fewest hypothesis testing rejections (78%), whereas PalA particles exhibited the fewest rejections (44%). Note, the goodness-of-fit test was merely used to quantify similarity to DP C’s pdf; less stringent tests or metrics can be considered. This and other issues, such as practical vs. statistical significance, are discussed elsewhere.^25^

**Figure 5.**
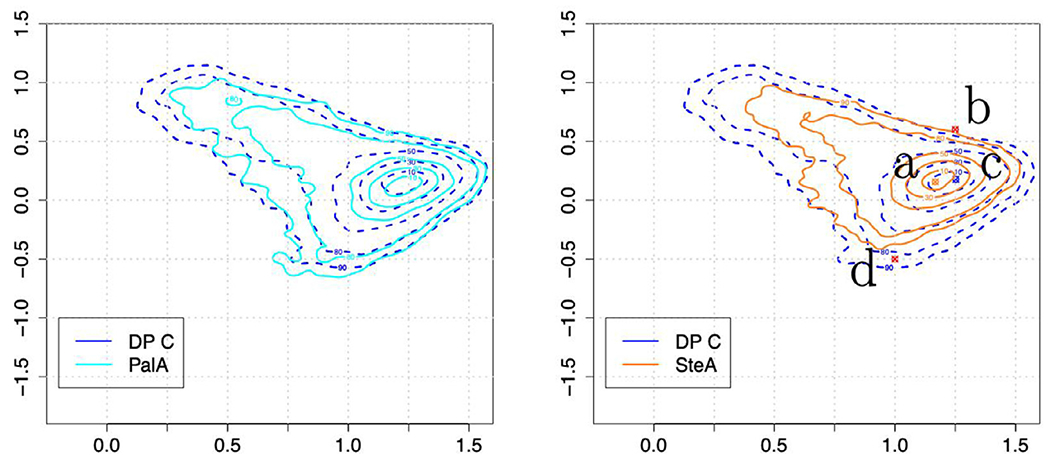
Comparison of fingerprints of particle images from PalA (left) and SteA (right) with DP C. Circles labeled “a” and “c” indicate the modes of the embeddings of SteA and DP C, respectively. The points labeled “b” and “d” mark two tail regions of SteA’s and DP C’s fingerprints.

**Figure 6.**
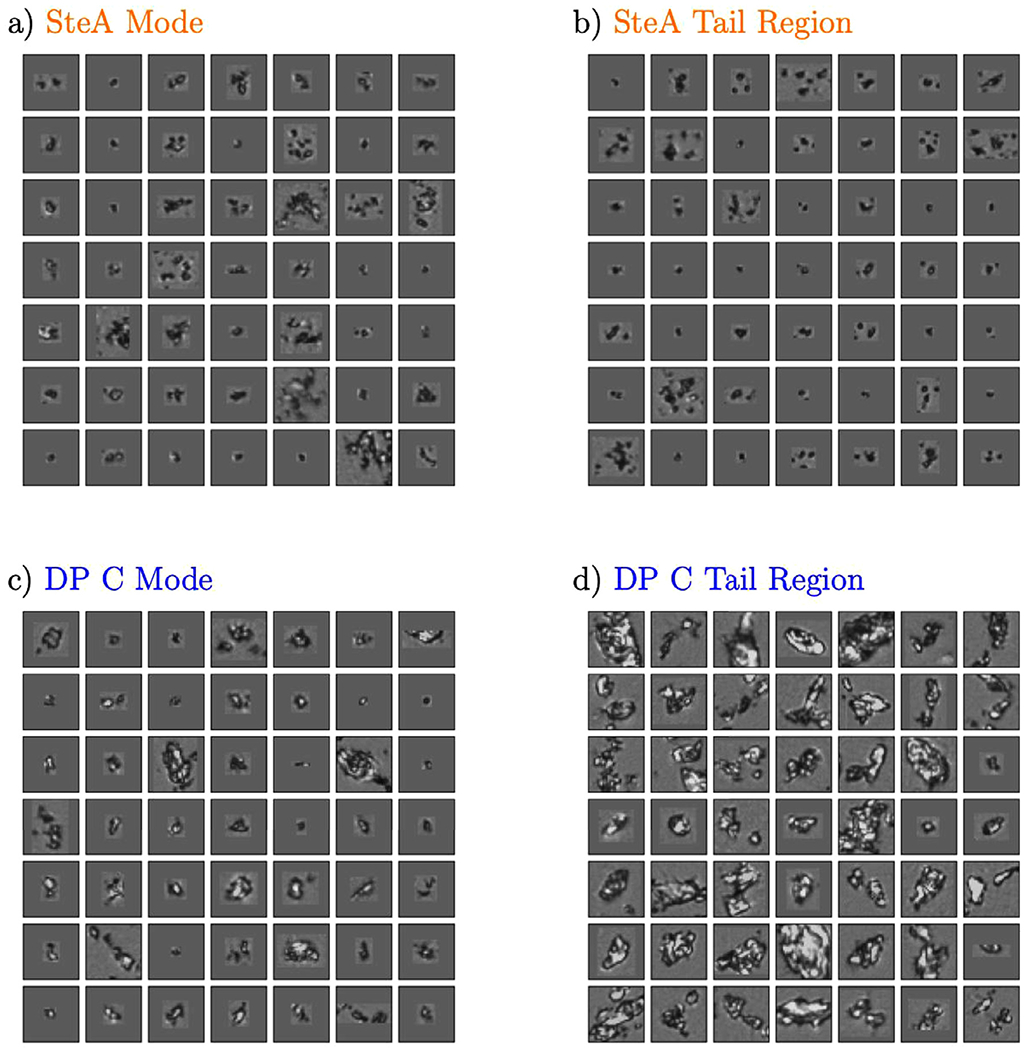
Source BMI images nearest the points shown in Fig. 5.

Figure 5 displays the fingerprints of the synthetic model particles of the two FAs most similar to the FA particles in DP C. Note that the BMI images exhibit slightly off-set modes of fingerprints for the particles in sample DP C (c) and for synthetic SteA particles (a). Sample images from two tail regions of DP C (d) and SteA (b) particles highlighted in Fig. 5 are shown in Fig. 6.

### Discussion.

The fingerprint analysis showed that the BMI images of synthetic model FA particles from longer chain FAs, SteA (C18) and PalA (C16), were most similar to images of the FA particles in DP C (the pdfs of SteA and PalA particle images overlapped heavily with the DP C pdf, see Fig. 5). Thus, the pure PalA and SteA particle samples were closer, in terms of the morphology imaged by BMI, to DP C particles than the particle sample containing a mixture of FA species (FA Mix I, see Fig. 3 and 4). Although the fingerprint analysis was consistent with the CNN classification results (Table 4), the latter was surprising as the FA Mix I sample was intended to mimic FA particles in DP C with regard to the total FA concentration and the FA composition as discussed below.

Fingerprints for PalA particles and for the particles in DP C spanned similar regions of embedding space, however, there was a subtle difference in their fingerprints that was detectable by the goodness-of-fit testing method employed. SteA particle images and the images of DP C particles exhibited not only similar particle morphologies in their overlapping regions, but also some distinct morphologies which were identified by the fingerprint analysis (as shown in Fig. 6). Furthermore, the fingerprint analysis comparing BMI images of FA particles in DP C and of synthetic model FA particles was not consistent with the results of the LC-MS characterization of the FA particles in DP C. LC-MS revealed that myristic acid was the most abundant FA (≈46% of total FA mass) in particles isolated from DP C, followed by palmitic acid (≈33%). Lauric acid was present at ≈11% followed by stearic acid at about 10%.

One possible explanation for the apparent differences between the results from the fingerprint analysis and LC-MS could be that the morphology of FA particles might be impacted by the particle formation process. FA particles in DP C formed in a continuous process over several months with the availability of different FA species changing over time with the progressing PS20 degradation process. For example, the enzymatic hydrolysis rate was reported to depend on the hydrophilicity of the carboxyester species and the specific enzyme.^34–36^ In contrast, during the generation of the synthetic model FA particles (obtained by spiking into aqueous buffer) the entire mass of FA is instantly released into the formulation resulting in a fast precipitation (hours to days) of poorly soluble FAs. Furthermore, studies by Cao et al. (2015)^4^ and Almendinger et al. (2021)^37^ illustrate the potential impact of additional factors like presence of protein or glass leachables (e.g., aluminum ions) on the FA particle formation process. Accordingly, a better adjustment of the composition of synthetic model FA particle samples with respect to, e.g., protein or metal ion content, might improve the morphological resemblance between model and real-life FA particles.

Additionally, it remains to be clarified how well the ratios of the differing FA species in the particles formed in the Mix I sample agree with the actual FA species distribution in particles in DP C determined by LC-MS. In the applied protocol for synthetic model particle generation, the concentration and composition of the 2-propanol-dissolved FA mixture was consistent with the total FA mass and the FA species distribution in the particles in DP C. Nevertheless, according to Doshi et al. (2015)^2^, longer chain FAs (e.g., C18, C16) exhibit lower solubilities than short chain FAs like lauric acid (C12). Thus, different FA species in Mix I might have exhibited a differing extent of precipitation leading to differences between targeted and actual FA composition of the synthetic model FA particles. Moreover, the polysorbate raw material itself used in the preparation of the Mix I sample needs to be considered as important source of additional free FAs.^16^

Nevertheless, it should also be considered that differences between fingerprint and LC-MS analyses may be expected because the image based fingerprint approach performs data analysis on a distribution of particles (each imaged individually) while LC-MS results represent an averaged FA composition obtained from a total mass of particles retained on a filter surface.

Overall, the fingerprinting results provide a hint that the present protocol for the generation of synthetic model FA particles by spiking of organic-solvent-dissolved FA into formulation buffer can serve as a simplified but not fully representative model particle system to study the formation of particles related to the degradation of polysorbate in DPs.

### Case Study 3: Comparison of BMI FA Particle Images Acquired Over Time in Laboratory Prepared Samples

**Figure 7.**
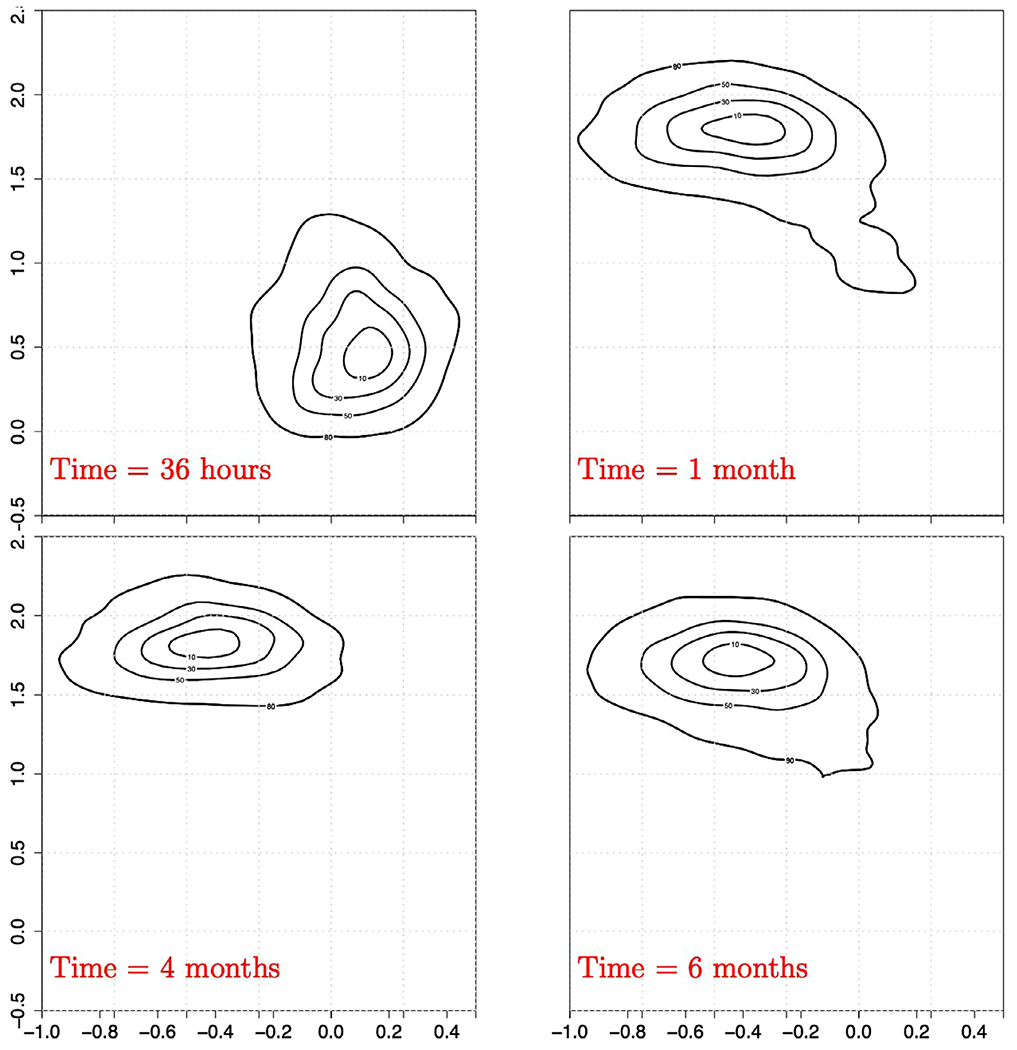
Fingerprints of the FA Mix III sample analyzed at four different time points (36 hours, 1, 4, and 6 months: from top to bottom).

**Figure 8.**
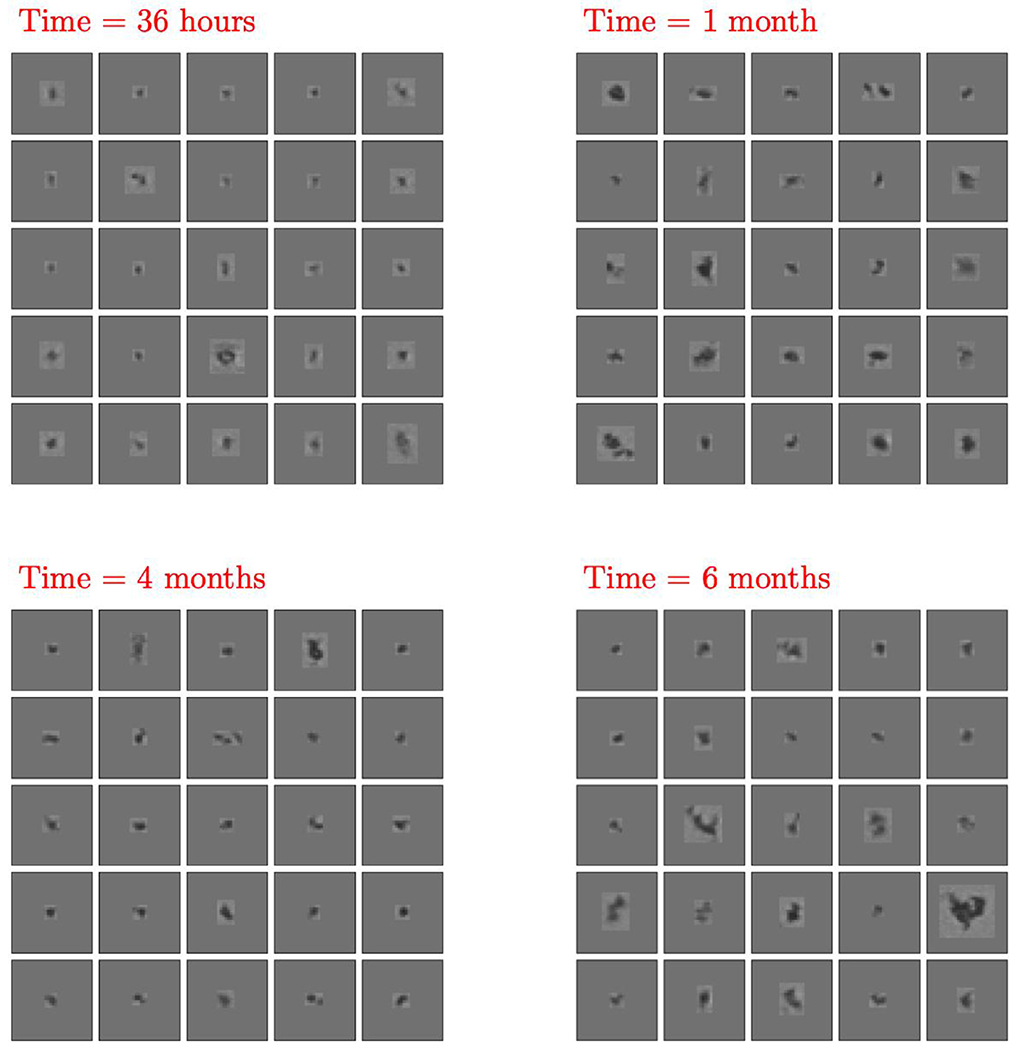
Source BMI images nearest the mode of the fingerprints shown in Fig. 7. The images represent FA particles in Mix III (at 36-hours, 1-, 4-, and 6-month time points).

In our third case study, BMI particle images acquired over 6 months from another synthetic model mixed FA particle sample (FA Mix III) were evaluated for relevant changes in particle concentration and morphological properties. The total number of particles continuously increased from ≈ 2,000 particles/mL ≥2 *μ*m at 36 hours, to ≈ 10,000 particles/mL ≥2 *μ*m at 1 month and reached ≈ 16,000 particles/mL ≥2 *μ*m at 6 months. For the particle morphology assessment, the same CNN embedding network architecture as stated above was used, but this time the network was trained with data from model particles in the FA Mix III sample mimicking FA particle formation in presence of salts (aluminum (III) chloride, NaCl; see Table 1). The network was trained using FA Mix III particle images from the same sample imaged at 36 hours, 1 month, and 4 months. The 6-months images were evaluated out-of-sample (i.e., they are not contained in the training class). Figure 7 displays the fingerprints obtained at the four different time points. In Fig. 8, we display images for the ≈ 50 closest (in terms of Euclidean distance) embedding points to the pdf modes shown in Fig. 7 for the 36-hours, 1-, 4-, and 6-months time points.

### Discussion.

Fingerprint analysis of the BMI images from FA Mix III particles revealed a meaningful change over time in particle morphology, i.e., a different fingerprint distribution and location for BMI particle images at 36 hours compared to all later time points (Fig. 7). In contrast, time points at 1, 4 and 6 months exhibit a common mode and shape in terms of the fingerprint distribution indicating no further meaningful morphological changes in imaged particles. Representative particle images in Fig. 8 confirm that there are subtle (although not easily detectable by human eye) morphological differences in the particles rapidly formed within 36 hours, i.e., the boundaries and interior of the particles are lighter, compared to those particles present in the same sample after longer storage times (≥1 month) where the particles appear more compact and darker. The change in morphology between particles imaged at 36 hours and ≥1 month might be explained by different phases during particle formation. The fact that after 1 month the particle morphologies remain similar suggests that the mechanism of particle formation remains the same and only more particles of the same morphology are formed after that time point.

Interestingly, Almendinger et al. ^37^ propose a two-stage model for the formation of FA particles in the presence of aluminum ions: in the initial phase, nucleation seeds are formed by complexation of aluminum ions and FA, in the second phase, additional FA molecules accumulate at the seeds, which finally results in precipitation of particles. Despite the fact that the authors suggest a change in growth mechanisms before the actual presence of subvisible particles, their hypothesis reinforces that FA particle formation might proceed in successive mechanistic steps. Although our results appear to be consistent with this hypothesis, additional work is needed to fully understand the particle formation mechanism.

### Classification and Comparison of BMI Particle Images in Comparable Samples

In previous data analysis the usefulness of fingerprinting to differentiate between particles of different origin or formed by different mechanisms was demonstrated. We also wanted to evaluate if BMI particle images of the same origin in comparable samples would be classified as similar by the fingerprinting approach. To this purpose, several vials of the same batch of DP C, each containing FA particles, were analyzed on the same day. In addition, frozen (−80°C) aliquots of the same DP A sample with protein particles were analyzed 2 months apart.

**Figure 9.**
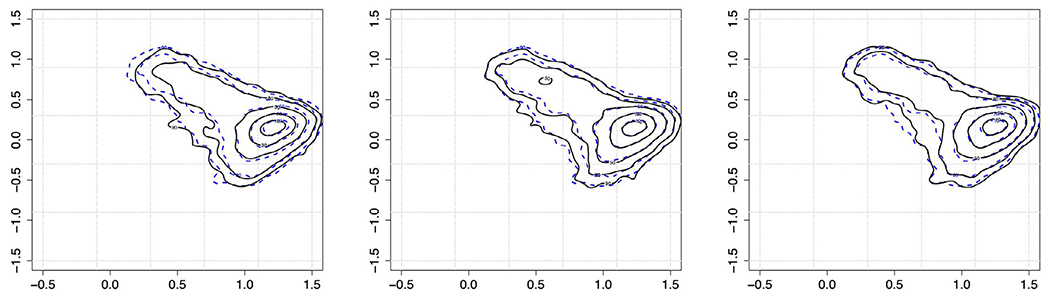
Fingerprints for FA particles from three separate vials of DP C. The different vials were from the same batch and of the same age. Fingerprints of the single vials are plotted in black along with the fingerprint of the entire DP C sample (consisting of data from all 3 vials) indicated as blue dashed line.

Fingerprints of BMI FA particle images (Fig. 9) from three separate vials of DP C analyzed on the same day demonstrate qualitatively that there is little variation between the embedding distributions. The fingerprint of the pooled data (blue dashed line) from the entirety of all imaged DP C particles is representative of each vial (plotted in black). Furthermore, using the entire DP C distribution (combining the particle images from all vials) as the null density, the quantitative goodness-of-fit rejection rates were 5%, 1%, and 7% for vials 1-3, respectively, showing that the distribution of embedding points was not inconsistent with the null density in the repeat vials.^1^

The standard CNN classifier did show comparable classification results for images of protein particles from frozen DP A analyzed 2 months apart (we refer to the later analysis run as DP A*), i.e., 84% of the BMI particle images from DP A* were correctly classified compared to 81% in the case of the initial DP A run. The fingerprinting approach also provides comparable BMI particle image classification results between both runs (data not shown).

### Discussion

BMI particle image analysis by CNN classification or by the fingerprinting approach provides consistent results for comparable samples with respect to particle origin or age. This was demonstrated by highly comparable embedding distributions (Fig. 9) for FA particles from different DP C vials of the same batch, which were analyzed side-by-side. Formal hypothesis testing failed to detect significant differences in these BMI imaged particles sampled from different vials. In addition, classification results for BMI images of (preserved) protein particles from two separate analytical runs of −80°C frozen DP A support that morphological similarities in BMI images are correctly and reproducibly assessed and differences meaningful when found between BMI images by both approaches.

## Conclusions

In conclusion, both traditional supervised CNN classifiers, as well as the recent fingerprinting approach^20^ demonstrated that images from BMI contain representative morphological features capable of distinguishing various particle types studied (e.g., images of different FA and protein particles can readily be distinguished from one another). Furthermore, we showed how the CNN based fingerprinting approach can be used to both qualitatively and quantitatively characterize morphological differences in particle populations (if particle classes exhibit resolvable morphological differences). The fingerprinting approach also enables the characterization of the morphological similarity of BMI particle image distributions of different particle types (e.g., DP A and FA Mix I particle images exhibited highly similar morphologies when analyzed as a population).

Previously, the fingerprinting approach was demonstrated as a promising tool in the field of particle analysis when combined with flow imaging microscopy.^20,24,25^ In this work, we demonstrated that extracted BMI particle images using proprietary software complemented by the fingerprinting approach can be a valuable label-free method for high-throughput particle classification and characterization. Particle classification and characterization based on morphological features, in addition to particle count and size, can be utilized for the monitoring of particle formation over the different stages of product development. Furthermore, our findings illustrate that the fingerprinting approach can help to gain new insights in studies aiming to mimic and understand particle formation mechanisms related to polysorbate degradation in drug products.

Future research and case studies will assist in elucidating the capability of particle classification using BMI complemented by data analysis based on CNNs or other AI. Finally, while today’s routine particle analysis instruments are optimized for sizing and counting of particles, it would be interesting to explore how fingerprinting and other CNN based AI perform when combined with imaging techniques optimized for image resolution (hence recording higher fidelity digital information about particle morphology).

## Supplementary Material

1

## Figures and Tables

**Table 1:** Overview of Samples and Properties. Additional Abbreviations Used here but not Explained in the Previous Text are: LauA (Lauric Acid, C12), MyrA (Myristic Acid, C14), PalA (Palmitic Acid, C16), SteA (Stearic Acid, C18) and OleA (Oleic Acid, C18:1).

Sample Designation	Sample Solvent	Concentration of Relevant Component	Particle Type
DP A (DP A*: second analytical run)	histidine buffer with 0.04% (w/v) PS80	≈50 mg/mL mAb 1	protein particles with no meaningful levels of other constituents
DP B	sodium phosphate buffer with 0.01% (w/v) PS80	≈20 mg/mL mAb 2	
DP C	histidine buffer with 0.04% (w/v) PS20	>100 mg/mL mAb 3	FAs or salts of FAs with no significant amount of protein
FA Mix I	DP C formulation buffer	22 *μ*g/mL (Mix I and Mix III) or 11 *μ*g/mL	mixed FA particles (artificially generated)
FA Mix II	DP C formulation buffer	(Mix II) of total FA: composed of LauA	
FA Mix III	DP C formulation buffer + 77 mM sodium chloride and 6 ppb aluminum (III) chloride, pH 5.0	(~55%), MyrA (~18%), PalA (~11%), SteA (~5%), and <5% each of OleA, caprylic, and capric acid (percentages as w/w of total FA)	
Single FA samples	DP C formulation buffer	10 *μ*g/mL of one FA (PalA or SteA or MyrA) or 40 *μ*g/mL of LauA	single FA particles (artificially generated)
ETFE	> 99% water + 0.02% sodium azide + 0.02% 4 (1, 1, 3, 3 tetramethyl butyl) phenyl polyethylene glycol	< 0.01%	ETFE

**Table 2:** Confusion Matrix Showing Ground-truth (rows) vs. Predicted Label (columns) for Four Different Classes of Particles: the Protein Surrogate ETFE, PalA (C16), and DP A and DP B. In Each Row, the Numbers Listed Correspond to the Fraction of Individual BMI Test Images Classified into the Category Labels Shown in the Columns). Green Color Highlights Classification Results for the True Class (a Perfect Classifier would Exhibit Ones Along the Diagonal). Pink Color Indicates Fraction of Misclassification.

	ETFE	PalA	DP A	DP B
ETFE	0.95	0.02	0.01	0.02
PalA	0.03	0.77	0.10	0.09
DP A	0.01	0.09	0.81	0.09
DP B	0.02	0.07	0.09	0.82

**Table 3:** Confusion Matrix of FA Particle Classes. The CNN was Trained with Images of a Large Collection of Particles from Pure FAs and FA Mixtures of Known Composition. See Table 1 for a Detailed Description of the Samples Analyzed. The Ground-truth Row Labels have been Omitted for Clarity (Top to Bottom Ground-truth Labels Correspond to Left to Right Column Labels). Color Code: Green Indicates Fraction of Correct Classification, Pink Marks Fraction of Misclassification.

C18	C16	C14	C12	Mix II	Mix I
0.64	0.26	0.00	0.00	0.00	0.09
0.20	0.67	0.03	0.00	0.02	0.09
0.00	0.03	0.87	0.07	0.03	0.00
0.01	0.01	0.11	0.60	0.27	0.01
0.00	0.01	0.02	0.20	0.75	0.01
0.06	0.06	0.00	0.01	0.03	0.80

**Table 4:** Classification of Particle Images from DP C Using the CNN from Table 3, in which DP C was not Used as a Training Class. Notably, Orthogonal LC-MS Analysis (See Methods Section) Indicated that Particles in DP C were Primarily Composed of FAs or Salts of FAs with no Significant Amount of Protein. Using BMI Images, the CNN Predicts PalA (C16, no Color) as the Most Likely Label when Analyzing ≈ 20k DP C BMI Particle Images. Pink Cells Indicate Particle Classes DP C Particles were less Frequently Assigned to.

C18	C16	C14	C12	Mix II	Mix I
0.25	0.51	0.03	0.00	0.01	0.20

## Footnotes

^1^ The reported quantitative goodness-of-fit rejection rates used an *α* = 0.05 and subsampled the data (using batch size of N = 100 particle image embedding subsets) to check for consistency with the null density.
