# Supplementary material for: Combining Machine Learning and Backgrounded Membrane Imaging: A case Study in Comparing and Classifying Different types of Biopharmaceutically Relevant Particles
Source: J Pharm Sci. Author manuscript; Available in PMC 2022 Sep 1. (PMC9391316; doi:10.1016/j.xphs.2022.05.022)
Supplement: 1 [file NIHMS1819971-supplement-1.docx]

**Supporting information**


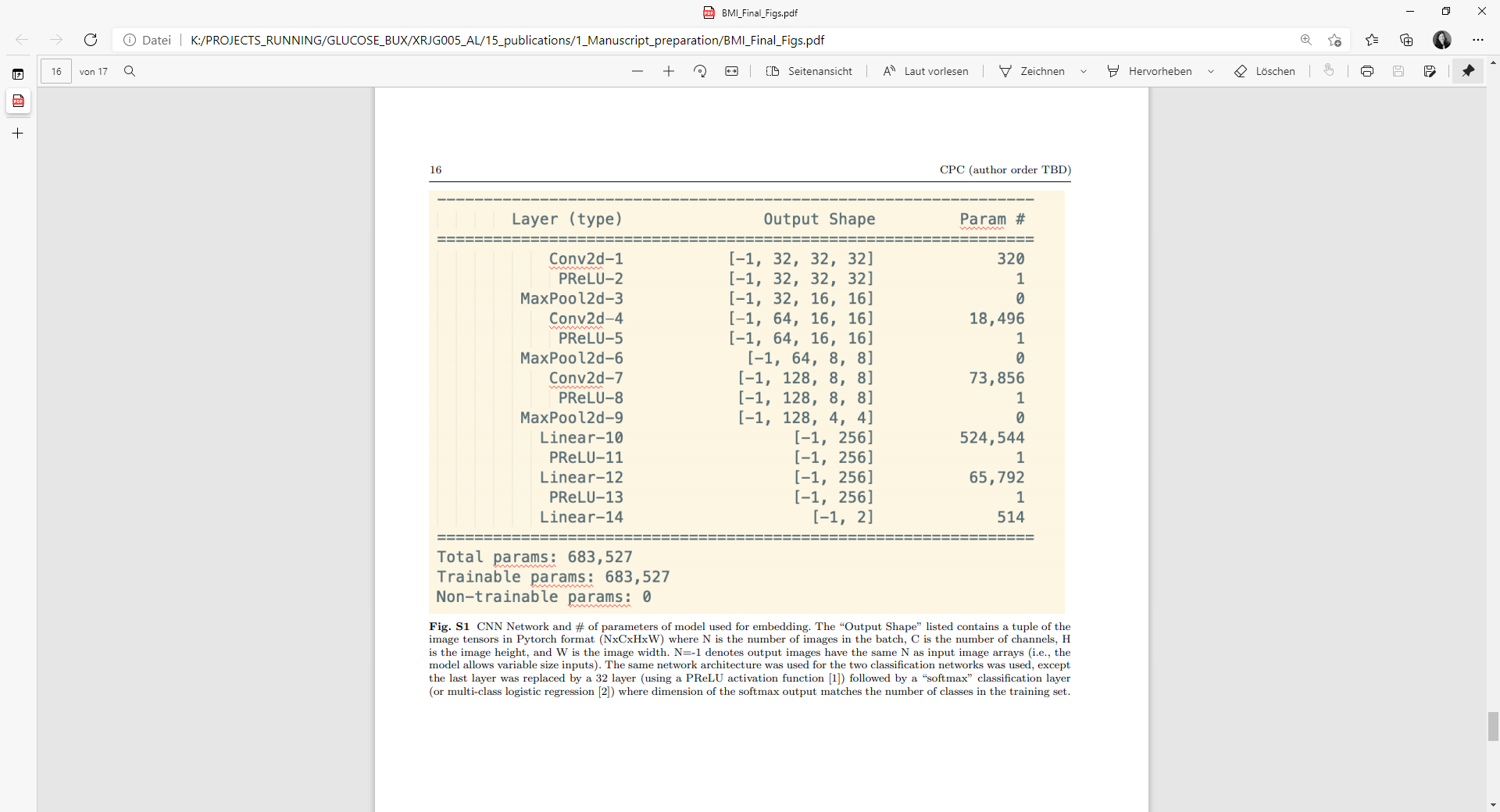


Fig. S1 CNN Network and # of parameters of model used for embedding. The “Output Shape” listed contains a tuple of the image tensors in Pytorch format (NxCxHxW) where N is the number of images in the batch, C is the number of channels, H is the image height, and W is the image width. N=-1 denotes output images have the same N as input image arrays (i.e., the model allows variable size inputs). The same network architecture was used for the two classification networks was used, except the last layer was replaced by a 32 layer (using a PReLU activation function [1]) followed by a “softmax” classification layer (or multi-class logistic regression [2]) where dimension of the softmax output matches the number of classes in the training set.


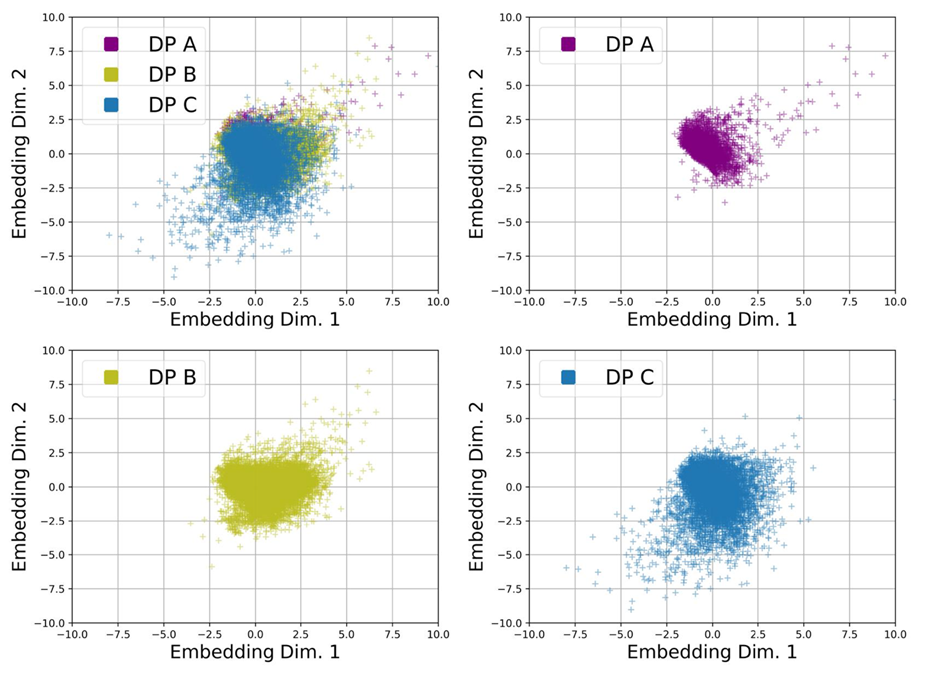


Fig. S2 Principal components analysis (PCA) embeddings of BMI images shown in Fig. 2a. Top left: PCA embeddings of DP A-C of the first two principal components.  The remaining panels show the individual drug products since the PCA embeddings overlap heavily.  Results were obtained using scikit-learn PCA package with whitening; results without whitening were qualitatively similar (i.e., high overlap still exists with DP A-C), but with different embedding axis scale.

Table S1. Results identical to Table 2 in main text, except the CNN was trained with 24x24 grayscale BMI images (i.e. the 32x32 images were further cropped).

|  | ETFE | PalA | DP A | DP B |
| --- | --- | --- | --- | --- |
| ETFE | 0.96 | 0.01 | 0.01 | 0.02 |
| PalA | 0.05 | 0.78 | 0.12 | 0.05 |
| DP A | 0.01 | 0.07 | 0.87 | 0.05 |
| DP B | 0.03 | 0.05 | 0.09 | 0.83 |


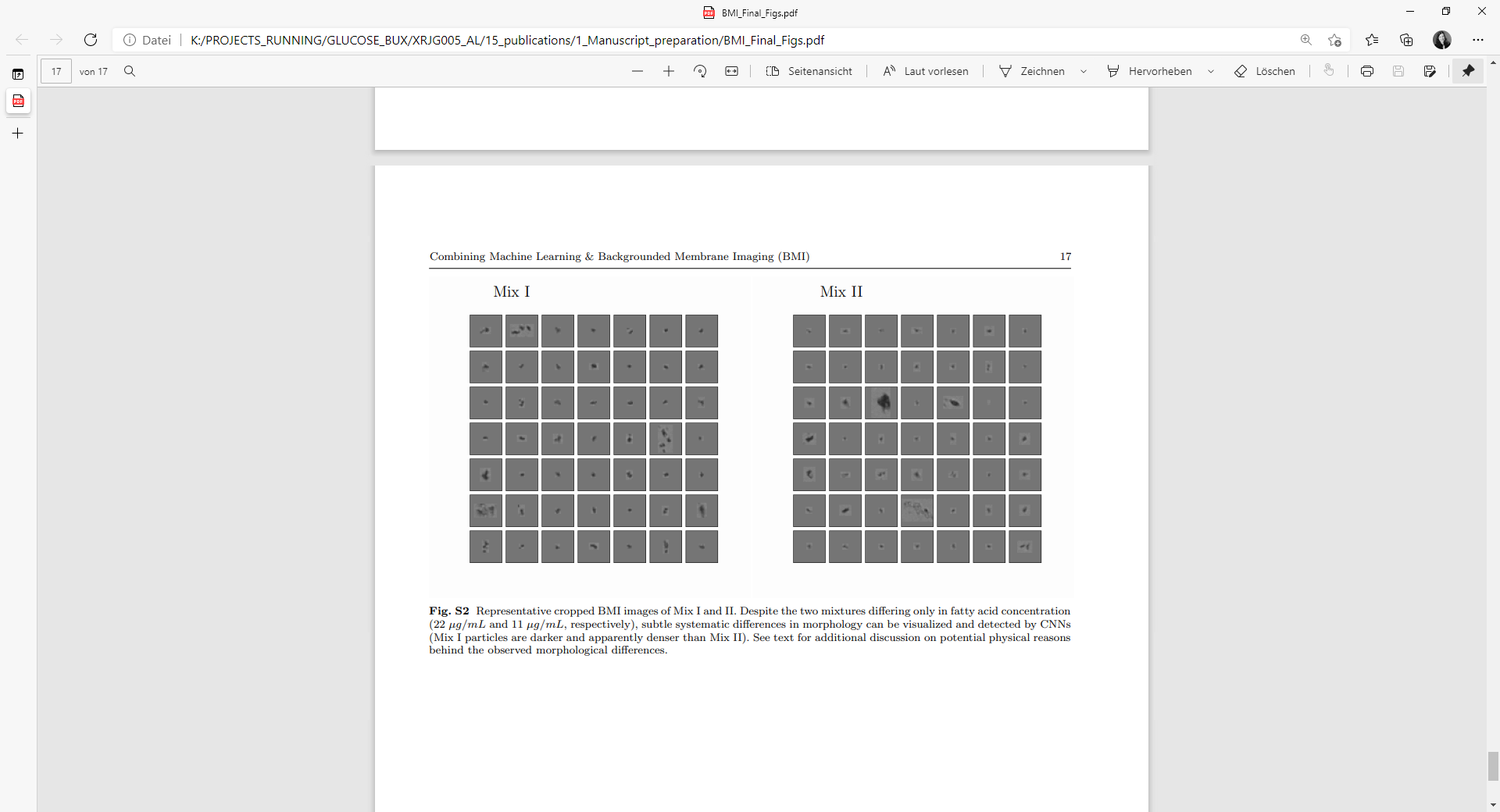


Fig. S3 Representative cropped BMI images of Mix I and II. Despite the two mixtures differing only in fatty acid concentration (22 µg/mL and 11 µg/mL, respectively), subtle systematic differences in morphology can be visualized and detected by CNNs (Mix I particles are darker and apparently denser than Mix II). See text for additional discussion on potential physical reasons behind the observed morphological differences.
